# Case of Hydrops Medullæ Spinalis, or What Is Commonly Called Spina Bifida

**Published:** 1809-01

**Authors:** N. Washbourn

**Affiliations:** Surgeon, and Member of the Royal College of Surgeons, London, Marlborough, Wilts


### *To the Editors of the Medical and Physical Journal.*

GENTLEMEN,

SHOULD the annexed case, accompanied with an accurate drawing* of the same, be found sufficiently deserving a place in your very useful periodical Publication, you will confer on me an additional obligation by its insertion.

---

* We are much obliged to Mr. WASHBOURN for the accompanying drawing; but as it did not seem necessary to illustrate the Case, we have omitted to engrave it. ED.

### *Case of Hydrops Medullæ Spinalis, or what is commonly called Spina Bifida.*

THE following is the first malformation of the kind which has fallen under my observation; how distressing, when it is considered, that every effort both of medical and chirurgical aid proves ultimately abortive!

The disease in question has generally been termed Spina Bifida, which may in my opinion be considered as the effect, and that the Hydrops Medullæ Spinalis appears, ab origine, to be the primary and proximate cause of this morbid affection.

About three months since I was requested by Mrs. Dixon, of this town, to give my attendance and opinion, relative to a swelling occupying the lumbar region of her female infant, who was then three days old, a very fine and healthy looking child.

The case on inspection was most obviously and decidedly a deficiency or want of two of the inferior spinous processes of the lumbar vertebra. Through the aperture projected a long sac or tumour, containing a diaphanous fluid, and which appeared to be an elongation of the dura mater. On making a gentle pressure with the hand upon the tumour, there was evidently a free communication between it and the ventricles of the brain, constituting the disease called Hydrocephalus Internus.

The head was large, and the fontenelles and different sutures of the cranium were preternaturally divided. The child from its birth had strabismus; and it did not appear to have suffered very much from the disease, and had the use of the lower extremities until about a month previous to its dissolution, which happened on the 1st instant (Dec.) at the age of about 13 or 14 weeks.

Although there was a perpetual exudation of a perfectly colourless fluid from the tumour, it gradually increased in magnitude till within a few days of its death, when small incipient ulcerations took place upon different parts of the tumour, accompanied with inflammation. The case being a hopeless one, I advised the use of soft emollient cataplasms, which appeared to be the best and easiest kind of application.

### Dimensions of the Tumour.

A line drawn across from its basis in a horizon tal direc tion from the superior part of the sac measured nearly 4 inches, the longitudinal direction measured 3½ inches.

Before the child was buried I examined the part, and found found the integuments were become corrugated and flat, and the contents quite evacuated.

I am, &c.

N. WASHBOURN,

Surgeon, and Member of the Royal College of Surgeons, London,

*Marlborough, Wilts, Dec.* 6, 1808.

